# Multivariate spatial feature selection in fMRI

**DOI:** 10.1093/scan/nsab010

**Published:** 2021-01-27

**Authors:** E Jolly, L J Chang

**Affiliations:** Computational Social Affective Neuroscience Laboratory, Department of Psychological and Brain Science, Dartmouth College, Hanover, NH 03755, USA; Computational Social Affective Neuroscience Laboratory, Department of Psychological and Brain Science, Dartmouth College, Hanover, NH 03755, USA

**Keywords:** multivariate, feature-selection, searchlight, biomarker, decoding, fMRI

## Abstract

Multivariate neuroimaging analyses constitute a powerful class of techniques to identify psychological representations. However, not all psychological processes are represented at the same spatial scale throughout the brain. This heterogeneity is apparent when comparing hierarchically organized local representations of perceptual processes to flexible transmodal representations of more abstract cognitive processes such as social and affective operations. An open question is how the spatial scale of analytic approaches interacts with the spatial scale of the representations under investigation. In this article, we describe how multivariate analyses can be viewed as existing on a spatial spectrum, anchored by searchlights used to identify locally distributed patterns of information on one end, whole brain approach used to identify diffuse neural representations at the other and region-based approaches in between. We describe how these distinctions are an important and often overlooked analytic consideration and provide heuristics to compare these different techniques to choose based on the analyst’s inferential goals.

## Introduction

The past decade has witnessed an explosion in empirical studies employing advanced statistical methods to understand brain representations. Traditional univariate analyses of functional magnetic resonance imaging (fMRI) data have historically focused on differences in magnitudes of activation (Friston *et al.*, 1995), while more contemporary approaches have explored how spatial patterns of activity encode psychological information (multivariate pattern analysis; MVPA) (Haxby *et al.*, 2014) and how the temporal dynamics of neural responses are shared across individuals (intersubject correlation; ISC) (Nastase *et al.*, 2019). Unlike univariate techniques that independently model each voxel, these modern techniques often involve aggregating responses across multiple voxels during the modeling process (e.g. searchlights, regions of interest (ROIs) or whole brain). An underappreciated consideration when using these approaches is the spatial scale at which these analyses are performed. In this article, we will discuss how different psychological and cognitive processes may be reflected at different spatial scales and how this might impact choices in the analysis pipeline. We begin by exploring evidence for spatial-scale heterogeneity, then compare and contrast the most commonly employed techniques and conclude with practical considerations for choosing methods best suited for different research questions.

### Spatial scale of representations in the brain

Many contemporary fMRI methods focused on mapping brain representations or modeling neural synchrony require selecting specific spatial features to be used in an analysis (e.g. fMRI decoding, encoding, representational similarity analysis (RSA), ISC, intersubject RSA (Naselaris *et al.*, 2011; Diedrichsen and Kriegeskorte, 2017; van Baar *et al.*, 2019; Chen *et al.*, 2019; Nastase *et al.*, 2019; Finn *et al.*, 2020)). In this context, features refer to the specific information that is entered into a model (e.g. a group of voxels, the average activity in a cortical region or a neural distance matrix) and used to make inferences about a specific process, representation or psychological state. Numerous published papers have made general recommendations about setting up and interpreting analyses with different techniques (e.g. Haynes, 2015). However, these guides primarily make recommendations based on statistical considerations such as the interpretability of decoding accuracy (Etzel *et al.*, 2013), or highlight what contemporary techniques offer beyond simple univariate contrasts of brain activity (Kriegeskorte and Bandettini, 2007).

A key consideration often missing from these discussions is the spatial variability with which different kinds of neural and/or psychological information may be represented in the brain (Kragel *et al.*, 2018). For example, considerable evidence stemming from neuronal recordings, univariate fMRI studies, neuropsychological investigations, computational modeling and animal studies has demonstrated a reliable functional organizational scheme for sensory systems, with a particular focus on the visual system (Felleman and Van Essen, 1991; Grill-Spector and Malach, 2004; Hubel and Wiesel, 2004; Yamins *et al.*, 2014). This modular organizational structure has served as a scaffold for much contemporary research and has also importantly impacted the analytic approaches used to make scientific discoveries. The structure of the visual system affords researchers the ability to test specific predictions and build models at fine spatial scales. Some notable examples include direct recordings of populations in preselected cortical patches (Chang and Tsao, 2017), or using local patterns of neural activity to topographically map how representations change and transform as information moves through the visual system (Kriegeskorte *et al.*, 2006). It has also been a key driver of highly sophisticated contemporary work such as comparing features learned by layers of deep neural networks to neural representations in different stages of the ventral visual stream (Kriegeskorte, 2015; Cichy *et al.*, 2016; Yamins and DiCarlo, 2016). This scale of analysis comports well with consensus understanding of how perceptual systems are organized and is well-suited for examining the brain through the lens of functional compartments or locally distributed populations of activity (Haxby *et al.*, 2014; Kragel *et al.*, 2018).

In parallel, a large body of work has taken a more macroscopic view of brain organization by examining how diffusely distributed representations and networks subserve different cognitive functions by dynamically adapting to the task at hand (Kragel *et al.*, 2018). At this spatial scale, cortical areas can be seen as belonging to various subtypes such as primary sensorimotor, unimodal associative, transmodal associative, paralimbic and limbic (Mesulam, 1998). These subtypes demonstrate independent patterns of functional connectivity at rest (rsfMRI) and can be used to parcellate the brain into distinct networks (Power *et al.*, 2011; Yeo *et al.*, 2011; Glasser *et al.*, 2016; Schaefer *et al.*, 2016). Interestingly, several groups have demonstrated that subtypes of cortex vary markedly in the similarity between their structural and functional connectivity (Honey *et al.*, 2009). For example, functional connectivity most closely resembles anatomical connectivity and microstructural properties in sensory and unimodal regions, but this resemblance breaks down in transmodal areas such as the default mode network (DMN) (Paquola *et al.*, 2019; Vázquez-Rodríguez *et al.*, 2019). Further, the variability in functional connectivity patterns appears to be organized around functional gradients that range from unimodal primary sensory regions to transmodal associative regions (Margulies *et al.*, 2016). In other words, neural activity at rest is organized in a manner consistent with the geometric structure of the brain. Brain regions farther away from primary sensory areas are responsible for less externally focused computations and more abstract modes of cognition (e.g. associative, multimodal and internally directed). Transmodal regions often exhibit less hierarchical organization, denser interconnectivity, more top-down projections between cortical layers and less laminar differentiation, which are believed to facilitate more abstract and flexible responding to different kinds of information (Paquola *et al.*, 2019; Vázquez-Rodríguez *et al.*, 2019).

The contrast between these domains serves to highlight the breadth of spatial scales at which the brain represents and supports different psychological and cognitive functions. If tight, localized, hierarchical organization of primary sensory systems represent one end of this range, the other appears to be a more spatially diffuse, abstract and flexible organization of transmodal areas. In the field of social and affective neuroscience, there appears to be a network of brain regions, overlapping with the DMN, thought to reliably support socio-emotional processing (Lieberman, 2007; Adolphs, 2009). An open question, however, is whether the functional organization of these regions resembles primary sensory systems with circumscribed functional subdivisions, or a more general structure such that all regions support socio-emotional cognition by flexibly adapting their responsibilities to the particular task at hand.

There is some evidence that this social brain network may contain distinct cortical areas, patches and populations of neurons with highly circumscribed responsibilities functionally tuned to specific aspects of a socio-emotional experience, akin to functional specificity in primary sensory systems (Adolphs, 2009). Meta-analyses of the medial prefrontal cortex (mPFC), for example, posit the existence of distinct subdivisions for cognitive and emotional tasks (Amodio and Frith, 2006; De La Vega *et al.*, 2016) and a dorsal to ventral gradient which delineates representations about others or the self, respectively (Mitchell *et al.*, 2006; Wagner *et al.*, 2012; Sul *et al.*, 2015). The temporoparietal junction (TPJ) has been strongly associated with theory of mind and specifically reasoning about others’ beliefs and intentions as distinct from their feelings and emotions (Saxe and Kanwisher, 2003; Peelen *et al.*, 2010; Young *et al.*, 2010; Carter *et al.*, 2012; Koster-Hale *et al.*, 2017), akin to the relationship between the fusiform gyrus and face processing (Kanwisher *et al.*, 1997). However, subdivisions within this area show different patterns of functional connectivity with the rest of the brain, suggesting distinct local representations despite cortical proximity (Mitchell, 2008; Mars *et al.*, 2012; Carter and Huettel, 2013). This work hints at a potentially fine-grained organizational structure within socio-emotional brain regions but has yet to be characterized to the same degree of functional and spatial granularity as primary sensory systems.

A different perspective proposes that socio-emotional representations might be more diffusely distributed because the phenomenological experiences themselves (e.g. feeling an emotion and inferring an intention) are by their very nature more abstract, consisting of the integration of numerous processes such as perception, memory, prediction, and interoception (Chang *et al.*, 2015; Barrett, 2017). Numerous studies support this account by demonstrating how regions within the DMN are critical for mental-state inference but also prospection, episodic memory, navigation, narrative comprehension, mind-wandering and high-level comprehension (Buckner and Carroll, 2007; Mason *et al.*, 2007; Spreng *et al.*, 2009; Simony *et al.*, 2016; Tamir *et al.*, 2016; Golchert *et al.*, 2017). A wide range of brain regions, spanning multiple networks, including the default-mode, salience, and frontoparietal, appear to be involved in the representation of emotions (Kober *et al.*, 2008; Lindquist *et al.*, 2012; Chang *et al.*, 2015; Wager *et al.*, 2015; Kragel and LaBar, 2016). Further, even local neural patterns within specific areas such as the anterior TPJ demonstrate flexible responding as the same neural populations encode information about distances in space, time, as well as social ties (Parkinson *et al.*, 2014) or are broadly involved in establishing social context (Carter and Huettel, 2013). In this view, socio-emotional representations are entangled with other cognitive processes because they depend upon them. As such, neural representations appear to be correspondingly diffuse, recruiting distributed dynamic brain networks that can flexibly represent the highly abstract nature of social and emotional experiences.

### What is the problem?

Given the heterogeneity of the spatial scale of different psychological processes, this immediately raises a question: how do the spatial scales of various analytic techniques interact with the representations they are measuring? For example, due to their inherently small spatial scale, searchlights are highly sensitive to identifying locally distributed patterns (Kriegeskorte *et al.*, 2008; Kriegeskorte and Diedrichsen, 2019), making them well suited to investigating representations that themselves are organized in a fine-grained manner (e.g. perceptual features). On the other hand, whole brain models, which jointly model functional responses across the entire brain, have been more successful than searchlights in identifying sensitive and specific predictive models of more abstract psychological processes such as pain (Wager *et al.*, 2013), negative affect (Chang *et al.*, 2015), guilt (Yu *et al.*, 2020), empathy (Krishnan *et al.*, 2016; López-Solà *et al.*, 2017) and identifying supramodal emotion categories (Kragel and LaBar, 2016). These examples raise the possibility that the efficient study of neural representations requires methods that coincide with the scale at which representations are organized. This problem is similar in nature to the choice of spatial smoothing kernel used in conventional fMRI analysis, whereby the optimal kernel size is dictated by the spatial extent of the hemodynamic response function as per the matched filter theorem (Friston, 2007). A large body of work has investigated how acquisition parameters like spatial resolution and pre-processing choices like smoothing affect the sensitivity of various analyses such as fMRI decoding (e.g. Gardumi *et al.*, 2016; Todd *et al.*, 2016; Yoo *et al.*, 2018). However, there have been far fewer studies investigating the optimal spatial scale (kernel size) of different multivariate analysis techniques (e.g. Stelzer *et al.*, 2014). This necessitates that researchers carefully consider the spatial scale of their analyses, rather than defaulting to particular pipelines. To aid in this process, we compare and contrast how common methodological conventions may interact with the spatial scale of neural representations.

### Current conventions

**Fig. 1.**
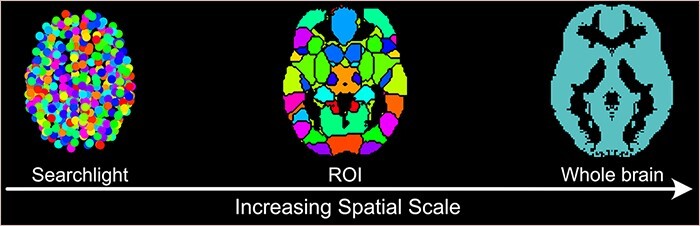
Spatial scales of different analytic strategies. Most common analytic methods can be seen as lying on a spectrum of varying spatial scales. Searchlights (left) represent one endpoint of this spectrum as they are well suited for modeling information at small spatial scales such as fine-grained neural patterns in a local neighborhood around a voxel defined by a radius size. ROI (middle) approaches can be used to model larger spatial scales explicitly taking into account functional and anatomical divisions. Multiple ROIs can be combined together to model even larger spatial extents such as functional networks. Whole brain (right) approaches represent the other endpoint of this spectrum as they are well suited for modeling diffuse representations that extend beyond local neighborhoods, regions and networks.

Whether researchers are performing MVPA analyses to test information encoding or decoding, ISC analyses to measure neural synchrony, or connectivity analyses to examine networks, each technique implicitly or explicitly constrains the spatial scale at which statistics are computed. Should separate statistical models be built for different voxels, neighborhoods or regions of the brain (i.e. independent groups of voxels)? And if so, how should this be determined? Should predictions, weights and variability from these models be combined to make inferences? And if so, how? Because different answers to these questions ultimately test very different statistical models, spatial feature selection becomes a key decision that always adds additional assumptions or constraints to the hypotheses being tested and the conclusions being drawn. Fortunately, there are numerous options available to researchers that fall along a spectrum of fine grain to diffuse spatial scales^1^ (Figure 1).

#### Searchlights.

The popular searchlight approach (Kriegeskorte *et al.*, 2006; Kriegeskorte and Bandettini, 2007) lies at one end of the spectrum and can be viewed as the ‘mass-multivariate’ analogue to the ‘mass-univariate’ approach popular in conventional activation-based fMRI analyses (Friston *et al.*, 1995). Searchlight analyses only consider information contained in local, overlapping neighborhoods around each voxel defined by a radius, and ignore how information may be distributed across spatial scales outside of those local neighborhoods. In this way, searchlights may ignore relevant signals in more diffuse representations such as emotions and are consistently outperformed by whole brain or regional models in those situations (Kragel *et al.*, 2018; Chang *et al.*, 2015). When used for decoding analyses, searchlights are equivalent to feature subset-selection in the machine-learning literature, whereby subsets are determined by the coordinates of each voxel and the radius of each searchlight (Hastie *et al.*, 2009). Similar to their univariate counterpart, searchlights are agnostic to functional or anatomical subdivisions and typically require as many statistical computations as voxels in the brain. Though rarely directly contrasted, searchlights can be easily compared as they are most often computed with the same radius size and therefore different searchlights contain the same number of voxels.

#### Regions of interest.

At a larger spatial scale, ROI approaches consist of groups of voxels determined by anatomical or functional divisions. There are broadly two types of ROI approaches: (i) contiguous and (ii) non-contiguous. Contiguous approaches consist of voxel groups that are spatially constrained to cover a continuous area of the brain, whereas non-contiguous approaches include both spatially contiguous but also spatially disjoint groups of voxels such as functional networks. Non-contiguous ROIs by their nature tend to encompass a larger spatial extent than contiguous regions. In both cases, spatial constraints are typically determined in two ways. One approach leverages functional responses, measured for example by using functional localizers from independent data (Saxe *et al.*, 2006) or by directly pruning voxels using techniques such as recursive feature elimination (De Martino *et al.*, 2008). The other approach relies on anatomical boundaries typically determined from brain atlases, rsfMRI connectivity network parcellations or meta-analyses (Yarkoni *et al.*, 2011; Chang *et al.*, 2013; De La Vega *et al.*, 2016; Eickhoff *et al.*, 2018; Shenton *et al.*, n.d.). The number of unique statistical computations estimated in the ROI approach is generally fewer than the searchlight approach and is determined based on the number of distinct regions selected. Unlike searchlights, ROI approaches can directly leverage known anatomical distinctions or functional response profiles as part of the spatial feature selection process. This flexibility enables them to capture a wide range of spatial scales, for example, modeling multiple distinct brain regions together or differentiating cortical sub-divisions across multiple models. More generally, ROI approaches are tests of focal hypotheses constrained to locations researchers often believe to be relevant a priori, such as social brain regions (Thornton and Mitchell, 2017). However, with this flexibility comes a trade-off in consistency across analyses. Comparisons across regions can become more complicated as ROIs typically don’t contain the same number of voxels.

#### Whole brain models.

Whole brain models reflect the largest spatial scale as they consider all voxels and their covariance during model estimation. In contrast to numerous small searchlights or ROIs, the whole brain approach can be viewed as a ‘single searchlight/region’ with a radius large enough to encompass all brain voxels. This approach can be used with unsupervised methods such as independent components analysis (Calhoun *et al.*, 2001; Beckmann *et al.*, 2005), or supervised methods such as decoding (Wager *et al.*, 2013; Chang *et al.*, 2015). Like searchlights, no anatomical information is explicitly used to determine the spatial scale of whole brain models. However, in decoding analyses, some more sophisticated algorithms can incorporate information about spatial smoothness or regional connectivity to find model estimates that better reflect the regional structure by forcing spatial constraints (Baldassarre *et al.*, 2012; Gramfort *et al.*, 2013; Grosenick *et al.*, 2013). Whole brain prediction analyses can provide a single model comprised of feature weights at each voxel that are simple to test in additional experimental contexts. Such generalization tests are highly valuable as they can provide valid reverse inference (Varoquaux and Poldrack, 2019) and also aid in identifying relative voxel importance (with caveats) (Haufe *et al.*, 2014; Kriegeskorte and Douglas, 2019). In addition, generalization tests can facilitate psychological construct validity, whereby model performance in different contexts can provide measurement information about the sensitivity and specificity of how a particular psychological construct is defined (e.g. different types of pain, memory and touch) (Kragel *et al.*, 2018). For this reason, these models have been particularly popular in translational and affective neuroscience, where whole brain decoders have been used as ‘biomarkers’ because they generalize well across populations and tasks even within a single subject (Wager *et al.*, 2013; Gabrieli *et al.*, 2015; Lindquist *et al.*, 2015; Krishnan *et al.*, 2016; Woo *et al.*, 2017; Kragel *et al.*, 2018).

## Analytic considerations

There are several key factors that researchers might consider when choosing between different scales of spatial feature selection. We have organized these factors into three broad categories. The first concerns subjective choices such as the goals of a particular analysis and the types of inferences researchers hope to make. The second comprises practical considerations for reliable statistical estimation. The third concerns computational resource availability and the trade-offs between different approaches. A summary of these comparisons is listed in Table 1.

**Table 1.** Comparison of different analytics strategies

	Searchlight	ROI	Whole brain
*Spatial Scale*	Fine-grained and fixed. Determined by searchlight radius which is typically the same for all searchlights.	Medium and flexible. Determined by how ROI was parcellated (e.g. functional responses, anatomy and network). Size reflects variable anatomy or functional response profiles.	Diffuse and fixed. Determined by sampling resolution of data (number of voxels).
*Conventional inferences*	Predictive performance of each searchlight (e.g. accuracy and correlation distance). Feature weights within searchlights typically not examined. Separate statistical models per individual and model performance aggregated at the group level.	Predictive performance for each ROI. Feature weights within ROIs highlight most informative voxels. Separate or common models across individuals.	Single predictive performance for model. Feature weights highlight most informative voxels. Separate or common models across individuals.
*Estimation (decoding)*	Independent models with overlapping features and some regularization (e.g. SVM). Anatomy is not part of estimation. *n > p; n ~ p; n < p*	Independent models with non-overlapping features and medium regularization (e.g. SVM and ridge). Anatomy can be used to define regions. *n < p; n ≪ p*	Single model that uses global covariance across all features with high regularization and/or dimensionality reduction (e.g. LASSO-PCR^a^) Anatomy is not part of estimation but provide constraints.^b^*n ≪ p*
*Compute Cost (CPU-time)*	High Large number of independent estimations required; more with permutation testing. Parallelization can reduce cost, but integrating results can be complicated	Medium Number of estimations depends on number of regions. Parallelization can reduce cost and integrating results is straightforward	Low Typically just one estimation and permutation regime performed. Parallelization is not trivial or not possible except for permutation testing or bootstrapping weights
*Compute Cost (Memory)*	Low/Medium memory Each searchlight has a small/medium memory footprint determined by radius and number of trials/conditions. Estimation rarely requires operating on all searchlight models simultaneously.	Medium memory Memory cost scales with the size of regions selected and number of trials/conditions/participants. Estimation rarely requires operating all ROI models simultaneously.	High memory Memory cost typically depends on total number of voxels (sampling resolution) and specific estimation routine (e.g. SVD). Estimation almost always requires operating on all voxels and observations simultaneously; exacerbated for between-subject models that require operating on many individual participants simultaneously
*Compute cost (Storage + Ease of Sharing)*	Low and simple if primarily working with performance only (e.g. accuracy maps, distance correlation) because each voxel is associated with a single value. High and complicated if intending to save feature weights because searchlights are overlapping. Data sharing typically consists of accuracy maps.	Low and simple because ROIs are most often non-overlapping and each voxel is associated with a single value (feature-weight or performance). Can represent performance and weight maps in a single standard format (array, NIfTI). Easy to apply to new datasets. Data sharing typically consists of accuracy maps, but feature weight maps are trivial to share as well.	Low and simple because just one model in which each voxel is associated with a single feature-weight. Can represent weightmaps in a single standard format. Data sharing typically consists of weight maps that are then applied to novel datasets

This table compares searchlight, ROI, and whole brain approaches in terms of their strengths and weaknesses along three categories: inferential goals, model estimation and computational resource demands. Legend: *n*: number of observations; *p*: number of features; *~*: approximately equal; *<* or >: less or greater than; *<<* or *>>*: much less or much greater than.

^a^ The number of dimensions of predictive group models is typically limited by the number of participants in the dataset.

^b^ See structured sparsity models (Baldassarre *et al.*, 2012; Gramfort *et al.*, 2013; Grosenick *et al.*, 2013).

### What is the goal?

A primary distinguishing factor between different analytic techniques is the type of inference researchers want to make. Broadly construed, modeling falls into two ‘cultures,’ (Breiman, 2001; Yarkoni and Westfall, 2016): inference emphasizes model interpretability and is evaluated using null-hypothesis-significance testing in a single context (e.g. a single dataset or task), while prediction emphasizes generalizability to new contexts and is evaluated based on out-of-sample model performance (Bzdok and Ioannidis, 2019). While this characterization cleanly distinguishes univariate magnitude-based analyses and multivariate predictive analyses, different multivariate analyses often conflate both goals in confusing ways (Hebart and Baker, 2018). For example, searchlight analysis was primarily conceived of as an information mapping technique and, when combined with cross-validated decoding, can approximate out-of-sample performance to make inferences about ‘where information is represented’ (Kriegeskorte *et al.*, 2006; Kriegeskorte and Bandettini, 2007). Decoding in the context of whole brain models has focused primarily on predictive performance and generalization to a variety of contexts such as developing brain–computer interfaces (Woo *et al.*, 2017; Hebart and Baker, 2018).

Reflecting these differences, results from searchlight analyses are typically reported as accuracy maps and inference is performed by comparing accuracy at each searchlight to empirical or permuted chance (Haynes, 2015) (Table 1 Conventional Inferences). However, the feasible conclusions that can be drawn from this approach only indicate whether at least one voxel in a local neighborhood is related to the outcome being predicted, not necessarily that every voxel in that neighborhood is reliably representing psychological information (Viswanathan *et al.*, 2012; Etzel *et al.*, 2013).^2^ Feature weights within a searchlight are almost never examined nor used to make predictions on completely distinct datasets. This is due to the fact that searchlights are most often overlapping, leading each voxel to have a different feature weight depending upon the particular searchlight (local neighborhood) it belongs to. This makes it infeasible to perform traditional feature importance testing (e.g. bootstrapping/permutation testing) as there are numerous possible ways to integrate these different weights across searchlights (e.g. see MIDAS (Varol *et al.*, 2018)). With increasing radius size, these issues make it nearly impossible to identify which voxels are most important for prediction, as accuracy scores are ‘smeared’ over spatial extents because searchlights are overlapping (Viswanathan *et al.*, 2012).^3^ Searchlight analyses are also often computed on individual brains and performance metrics (e.g. accuracy) are aggregated at the group level to draw inferences (Stelzer *et al.*, 2014). This also means that the particular geometry of a representation (i.e. the spatial layout of feature weights within a local neighborhood) is likely to differ across individuals, greatly complicating what types of valid group inferences are possible. Unlike univariate activation analyses, rejecting the null-hypothesis of conventional parametric tests on accuracies (e.g. one-sample *t*-test) only suggests that some individuals demonstrate an effect not that the effect is typical in the population (Nichols *et al.*, 2005; Stelzer *et al.*, 2013; Allefeld *et al.*, 2016).

In contrast, whole brain analyses are often concerned with generalization to completely new datasets, which can be comprised of different individuals (Woo *et al.*, 2017). While predictive performance is essential in translational applications, the resulting feature weights at each voxel also provide some useful information as to the spatial layout of the representations e.g. ‘neural signature’ (Wager *et al.*, 2013). Feature importance (Table 1 Conventional Inferences) can be assessed by thresholding via resampling methods such as bootstrapping or permutation (Stelzer *et al.*, 2014; Chang *et al.*, 2015); however, the resulting thresholded maps must be interpreted with caution. Unlike univariate activation maps, reliable weight maps do not indicate that a voxel explicitly represents psychological information but that in concert with other voxels it can effectively predict an outcome (Haufe *et al.*, 2014). In other words, some voxels may indeed represent outcome-relevant information, but some may serve to denoise other voxels which share correlated noise (Kriegeskorte and Douglas, 2019).

ROI analyses are flexible enough to inherit the strengths and weaknesses of both searchlight and whole brain analyses depending on the details of an implementation. Separate models can be estimated for disjoint ROIs and aggregated to make predictions, similar to kernel learning in machine learning, where different kernels are used for different regions (Filippone *et al.*, 2012; Schrouff *et al.*, 2013). A single model encompassing multiple disjoint voxels can also be estimated to draw inferences about a network of regions or voxels that share similar functional response profiles, e.g. ‘social-brain mask’ (Thornton and Mitchell, 2017). Because ROI methods don’t typically involve overlapping features like searchlights, accuracy maps do not suffer from spatial ‘smearing,’ and feature weights can be examined for relative voxel importance similar to whole brain models (Chang *et al.*, 2018). At the same time, performance metrics and generalization tests on separate datasets and contexts are feasible and straightforward, permitting inferences about the sensitivity and specificity of representations within single brain regions (Chang *et al.*, 2015; Krishnan *et al.*, 2016).

Thus, each end of the spectrum varies in its inferential goals. Searchlight decoding permits spatial inference based on isolated local neighborhoods tested in similar contexts while ignoring how that information is represented (ignoring feature weights) unless explicitly modeled with approaches like RSA. Because they are typically estimated separately across individuals, they do not identify shared or common representations, but rather whether any kind of task-relevant representations exist in the brain (Allefeld *et al.*, 2016). Whole brain models permit strong inferences about generalization, based on model performance, and diffuse inferences about the spatial location of representations based on feature weights. Most often in practice, whole brain models aim to learn a common representation that generalizes across individuals. Regional approaches land in-between these endpoints based on their particular implementation. All methods, however, can extend beyond simple decoding analyses to facilitate stronger inferences. Searchlight analyses can use cross-validated RSA or pattern-component modeling (PCM) with model comparison to test hypotheses about what stimulus features geometrically organize information within a neighborhood (Nastase *et al.*, 2017; Kriegeskorte and Diedrichsen, 2019). Different whole brain feature weight maps can be compared within the same context to determine representational specificity, share information and facilitate valid reverse inferences (Krishnan *et al.*, 2016; Varoquaux and Poldrack, 2019).

### Model estimation

The most common multivariate^4^ fMRI analyses are typically decoding models and RSA (Kriegeskorte *et al.*, 2006; Norman *et al.*, 2006). In decoding approaches, voxels are considered features, while time-points, trials, individuals or sessions serve as observations. Building a statistical model (e.g. a classifier, regression) requires estimating weights for features that can be combined to predict an outcome that generalizes over observations, such as properties of a task/stimulus (e.g. condition or category labels) or responses from individuals (e.g. behavior and emotional ratings).^5^ Voxel-selection procedures are the primary determinant of inputs that a statistical model uses to predict an outcome. This means that successful statistical estimation is heavily affected by the ratio between the number of features (*p*) and number of observations (*n*) (Hastie *et al.*, 2009). When *n *≥* p*, (more or equivalent observations than features) a model can be consistently^6^ estimated without further constraints. However, situations where *n* < *p* (fewer observations than features) yield a statistically underdetermined problem such that many unique combinations of features weights can yield the same predicted outcome. This issue is further exacerbated by the degree of independence between features. For example, spatial smoothing is a preprocessing step that can help boost signal-to-noise ratios but decreases spatial independence. Together these issues can lead to models that exhibit overfitting,^7^ whereby feature weights reflect both true signal but also idiosyncratic noise and generalize poorly to new data. To combat these issues, most estimation routines rely on some form of regularization, whereby constraints or penalties are used to limit the range of possible estimated feature weights. Common approaches include minimizing the squared (ridge and L_2_ penalty) or absolute magnitude (lasso and L_1_ penalty) (Hastie *et al.*, 2009) of feature weights. In many cases, these penalization techniques are similar to imposing differently shaped priors in Bayesian models (James *et al.*, 2013; Nunez-Elizalde *et al.*, 2019).

Since searchlights focus on local neighborhoods, their radius size, along with the details of an experimental task (e.g. number of conditions, trials, trials per condition, etc.), determine the ratio between features (voxels) and observations (trials, conditions) (Table 1 Estimation). Small neighborhoods comprise few features (e.g. ~28 voxels in a 6 mm radius searchlight collected at 2 mm voxel resolution volume) meaning approximately equivalent number of observations and features (*n *~ *p*) or a smaller imbalance of more features than observations (*n *<* p*; e.g. 100 voxels to 80 observations (Nastase *et al.*, 2017)). This may facilitate algorithms that require less regularization as evidenced by the popular use of linear models (e.g. linear discriminant analysis and support vector machine (SVM)) that exhibit good performance using default or variance-scaled hyperparameters rather than optimal hyperparameters tuned via cross-validation (e.g. Norman *et al.*, 2006; Hanke *et al.*, 2009). However, radii are often arbitrarily chosen based on sizes in previous studies and can have large effects on this ratio and thus may require different statistical models and regularization strategies, e.g. cross-validated MANOVA (multivariate analysis of variance) (Allefeld and Haynes, 2014). In addition, multiple comparisons corrections are needed to adjust for the large number of estimated models (Etzel *et al.*, 2013).

Since whole brain models include all voxels and are often used to identify representations that generalize across individuals, the features greatly outnumber observations (*n* ≪ *p*; e.g. 350k voxels to 182 individuals (Chang *et al.*, 2015)) often requiring stronger regularization (Kragel *et al.*, 2018) (Table 1 Estimation). For this reason, several studies use rigorous nested cross-validation along with independent hold-out sets to first tune regularization hyperparameters, then evaluate cross-validated predicted performance and, finally, test generalization performance on completely new individuals (Wager *et al.*, 2013; Chang *et al.*, 2015; López-Solà *et al.*, 2017; Kragel *et al.*, 2018). Another popular regularization approach is the LASSO-PCR (LASSO principal components regression), in which dimensionality reduction over all brain voxels is first performed using principal components analysis (PCA)^8^ followed by a sparse regression model (LASSO) to estimate weights on each principal component that are later inverted back into voxel space (Wager *et al.*, 2011). This approach jointly considers large groups of voxels with similar responses as single features used for prediction and produces sparse weight maps where only a few such voxel groups contribute strongly to prediction.

As noted in the previous section, the flexibility of ROI approaches, and the particular implementation chosen, will largely dictate the properties of an estimation regime. However, using a particular implementation such as non-overlapping, but contiguous ROIs, it may be possible to balance the strengths and weakness of both searchlight and whole-brain approaches (e.g. smaller neighborhoods, necessitates less regularization, but with estimable feature importance maps that can be used for generalization testing).

### Computational resources

The differences in inference and estimation routines between different techniques also impose different demands on computational resources (Table 1 Compute Cost). Broadly speaking, resources can be divided into three categories: (i) central processing units (CPU) time—the number of independent estimations required, the time required for each and the serial or parallelizability of the estimations; (ii) random access memory (RAM)—the ‘temporary’ working memory required to perform each estimation, typically determined by how and whether a particular algorithm needs to operate on all features and observations together, or can operate on them in a piecewise (batch) fashion and (iii) Storage—the hard disk space required to store the outputs of an estimation routine and the format of this storage that can determine ease of sharing models.

At the small spatial scale end of the spectrum, searchlights often demand high CPU costs, low to medium memory and, most often, low storage. This is because searchlight analyses require estimating as many models as there are voxels in a dataset. However, estimations can proceed in parallel and because features come from local neighborhoods with a small number of voxels, memory demands are typically low as well. Memory demands increase monotonically with increasing features and/or observations, i.e. larger radius or more task trials/conditions. If inferences are primarily made using accuracy maps, then storage is simple as a single value can be stored at each voxel location which can be easily shared. However, if researchers intend to store feature weights for each searchlight, storage becomes more complex due to large demands on disk-space and complicated indexing assigning feature weight vectors to each voxel location.

At the large spatial scale end of the spectrum, whole brain models often demand low CPU costs, high memory and low and simple storage. Because all voxels are used for estimation, only a single model needs be computed. However, because algorithms require operating on all voxels and observations simultaneously, they must hold and manipulate very large matrices (e.g. whole brain covariance matrix of 3k observations (100 participants with 30 trials each) by 200k voxels) in memory. Storage costs are low and straightforward as a model consists of a single scalar performance score and each voxel is only associated with a single feature weight, making whole brain models very easy to share and test on new datasets.

As with other analytic considerations, ROI approaches typically fall between searchlight and whole brain analyses with relatively medium CPU costs and memory but simple and low storage requirements. CPU costs can be minimized using parallelization like searchlight analyses. Memory demands scale with the size of each ROI as larger regions (e.g. non-contiguous DMN mask) require manipulating more features and observations together. Since ROI models are typically non-overlapping, they share storage demands similar to whole brain models as feature weights from different regions can be stored together in a single file along with binary masks to later extract the weights and apply them to new data. Accuracy maps derived from ROI models are similar to those estimated from searchlights, as only a single value needs to be associated with each voxel location.

For all spatial scales, cross-validation or non-parametric inference using resampling methods such as bootstrapping and permutation testing, will dramatically increase CPU costs and can potentially increase memory or storage requirements. This is because resampling methods require re-estimating a completely new model for each cross-validation fold and bootstrapped/permuted iteration. In the case of cross-validation or permutation testing, only the performance of each iteration needs to be retained, keeping storage costs low. However, bootstrapping distributions of feature weights requires retaining each iteration in order to define upper and lower uncertainty bounds (e.g. confidence intervals), thereby increasing costs depending upon researchers’ goals. For example, keeping feature weights in memory can reduce storage costs at the expense of increased RAM and decreased analytic flexibility down the line (e.g. loading and estimating a distribution). Saving feature weights to disk, on the other hand, increases storage costs by a factor of bootstrap iterations (each iteration produces a new set of feature weights of the same shape and size as the original model) but provides more analytic flexibility later on.

## Conclusions and recommendations

In this article, we have highlighted literature demonstrating how neural representations can exist at multiple spatial scales across the brain. Representations related to perceptual processes are often localized to small neighborhoods with highly specific response properties and hierarchical organization. Representations related to more abstract modes of cognition like social and emotional processing have been observed at fine spatial scales but more often consist of diffuse spatial representations spanning multiple regions and networks. This representational heterogeneity can interact with the spatial scale of particular analytic techniques, ranging from fine-grain pattern sensitivity in local neighborhoods (searchlights), focal tests of specific regions and networks (ROI), to whole brain neural markers that generalize across experimental contexts.

**Fig. 2.**
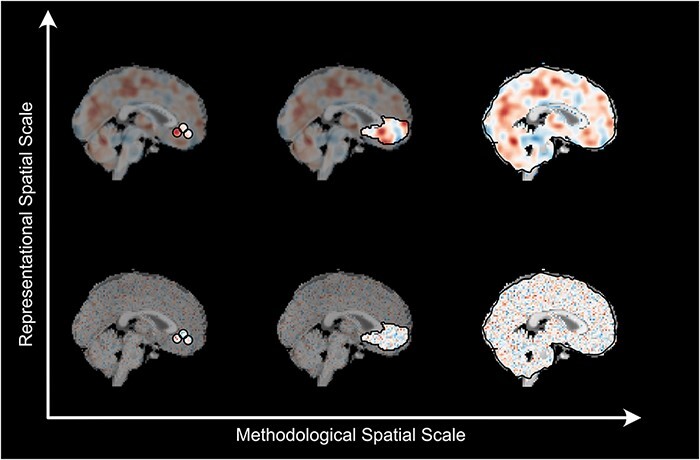
Interactions between methodological and representational spatial scales. Depending on the type of phenomenon under inquiry some analytic techniques may be more or less optimal. Increasing spatial scale of analysis techniques are depicted on the *x*-axis with searchlights at the small (left) end and whole brain approaches on the large (right) end; these mirror the spectrum Figure 1. The *y*-axis depicts hypothetical endpoints of representational scales with fine-grained local patterns in the bottom row (e.g. perceptual processes) and more diffuse patterns in the top row (e.g. social and emotional processes). Fine scale methods like searchlights may fail to capture diffuse representations as local neighborhoods provide a distorted view of a diffuse representation (top-row; left). These same methods may be optimal for finer neural representation in which all relevant information is reflected in a local neighborhood (bottom-row; left). On the other hand, large-scale methods such as whole brain approaches may be unable to reliably identify informative voxels when representations are organized in local neighborhoods (bottom-row; right) and may be better suited to identifying diffuse representations with large spatial extents (top-row; right). ROI approaches (top/bottom-row; middle) offer a flexible compromise, inheriting both the strengths and weaknesses of searchlight and whole brain approaches depending on the particular ROI method employed. At the same time, the smallest spatial scale measurable by fMRI is likely limited by the BOLD point-spread-function at a particular magnetic field strength, e.g. 3–5 mm at 3T (Parkes *et al.*, 2005).

While it may be tempting to iterate over many possible analyses and attempt to ‘optimize’ for the ‘best’ spatial scale, we caution researchers against framing the issue in this way given the lack of research specifically addressing this issue. For example, techniques like model comparison between searchlights and whole brain models are not trivial or even feasible to perform in most cases. Whole brain approaches estimate a single model, but other approaches estimate *N* models, where *N* is the number of ROIs or searchlights. Which of the *N* models should be used to compare to the whole brain model? Or should *N* models be combined into an ensemble? And if so how? One possible approach illustrated by Chang *et al.* (2015) (Supplementary Figure S4 Panel B) and Kragel *et al.* (2018) (Figure 2) compares the performance of whole brain models to the entire distribution of searchlight models but is unable to directly compare how different model weights capture the representation of emotions. Adding decision points to analysis pipelines without cross-validation multiplies analytic flexibility and will likely increase experiment level false-positive rates or facilitate ‘*p*-hacking’ (Carp, 2012). Instead, we recommend researchers more carefully select their analytic approach using a combination of empirical goals, estimation techniques and computational resources to determine what makes the most sense for the investigation at hand. At the same time, we believe the field may benefit from investigations directly examining the spatial scale of psychological phenomena thereby bringing greater clarity and more progress to this understudied issue.

## Footnotes

1 Because all methods fundamentally operate on information contained in voxels, fine-grained in this context refers to information in voxel patterns comprised of small (often-contiguous) spatial neighborhoods, whereas diffuse refers to voxel patterns encompassing much larger contiguous or non-contiguous spatial extents.

2 This same criticism does not necessarily apply to searchlight-RSA or searchlight-ISC analyses.

3 Smearing, however, can happen in principal with searchlight-RSA and searchlight-ISC analyses.

4 While encoding models can also be viewed as a kind of multivariate model, they are most often multivariate in stimulus feature space but univariate in brain space. In other words, high-dimensional models are primarily used to fit and predict a single voxel’s responses rather than a local or global spatial pattern (Nishimoto *et al.*, 2011; Huth *et al.*, 2016).

5 This delineation doesn’t perfectly capture RSA analyses as models are typically distance matrices derived from stimulus or task features and outcomes are neural distance matrices based on responses to those stimulus or task features.

6 Consistently here refers to a single solution (weights) that maps between features and outcomes conditional on some error/loss function (e.g. sum-of-squared errors/L_2_ norm in linear regression).

7 Underfitting is also possible, whereby feature weight fails to capture the true signal in a data, but occurs less often in fMRI analyses. This is because in most datasets, irrespective of spatial scale, researchers rarely have more observations being predicted (e.g. trials, conditions and individuals) than features used to make predictions (e.g. voxels), i.e. *n* < *p* or *n* << *p*.

8 In practice, the maximum number of retainable components is limited to the number of observations, typically individuals, in the dataset.

## Open science statement

A preprint of this manuscript has been submitted to bioRxiv.
